# Supplementary material for: Complexome profiling of the Chlamydomonas psb28 mutant reveals TEF5 as an early PSII assembly factor
Source: Plant Cell. 2025 Mar 17;37(6):koaf055. doi: 10.1093/plcell/koaf055 (PMC12164590; doi:10.1093/plcell/koaf055)
Supplement: koaf055_Supplementary_Data [file koaf055_supplementary_data.zip › psb28_interactive.zip!/plots/intensity/qualityPlots/peptides/_Cre01.g045550.t1.2.html]

### Cre01.g045550.t1.2

Trivial name:   
SeqCoverage [median]: 16.70  
Mass: 42.99 kDa  
  
 

| Sequence | MissedCleavage | Mass [Da] | wtIntensity | muIntensity | Score |
| --- | --- | --- | --- | --- | --- |
| AAIANVGASR | 0 | 928.51 | 104734.04 | 111207.01 | 196.88 |
| APPPPAAAGGASPAAA | 0 | 1272.65 | 2760.53 | 6047.58 | 130.75 |
| ELDALETTVSK | 0 | 1204.62 | 71240.22 | 77042.80 | 222.28 |
| KAPPPPAAAGGASPAAA | 1 | 1400.74 | 17528.07 | 19680.21 | 130.56 |
| SWSFGRPVTK | 1 | 1163.61 | 2409.83 | 3407.56 | 53.33 |
| TLEPAFNVVLSK | 0 | 1316.73 | 57414.79 | 53811.48 | 147.74 |
|  |  |  | 256087.5 | 271196.6 |  |
